# Motile bacteria leverage bioconvection for eco-physiological benefits in a natural aquatic environment

**DOI:** 10.3389/fmicb.2023.1253009

**Published:** 2023-12-13

**Authors:** Francesco Di Nezio, Samuele Roman, Antoine Buetti-Dinh, Oscar Sepúlveda Steiner, Damien Bouffard, Anupam Sengupta, Nicola Storelli

**Affiliations:** ^1^Department of Environment, Constructions, and Design, Institute of Microbiology, University of Applied Sciences and Arts of Southern Switzerland (SUPSI), Mendrisio, Switzerland; ^2^Department of Plant Sciences, University of Geneva, Geneva, Switzerland; ^3^Alpine Biology Center Foundation, Bellinzona, Switzerland; ^4^Department of Surface Waters – Research and Management, Swiss Federal Institute of Aquatic Science and Technology (Eawag), Kastanienbaum, Switzerland; ^5^Civil and Environmental Engineering, University of California, Davis, Davis, CA, United States; ^6^Faculty of Geosciences and Environment, Institute of Earth Surface Dynamics, University of Lausanne, Lausanne, Switzerland; ^7^Department of Physics and Materials Science, Physics of Living Matter Group, Luxembourg City, Luxembourg

**Keywords:** bioconvection, meromixis, Lake Cadagno, sulfur bacteria, eco-physiology

## Abstract

**Introduction:**

Bioconvection, a phenomenon characterized by the collective upward swimming of motile microorganisms, has mainly been investigated within controlled laboratory settings, leaving a knowledge gap regarding its ecological implications in natural aquatic environments. This study aims to address this question by investigating the influence of bioconvection on the eco-physiology of the anoxygenic phototrophic sulfur bacteria community of meromictic Lake Cadagno.

**Methods:**

Here we comprehensively explore its effects by comparing the physicochemical profiles of the water column and the physiological traits of the main populations of the bacterial layer (BL). The search for eco-physiological effects of bioconvection involved a comparative analysis between two time points during the warm season, one featuring bioconvection (July) and the other without it (September).

**Results:**

A prominent distinction in the physicochemical profiles of the water column centers on light availability, which is significantly higher in July. This minimum threshold of light intensity is essential for sustaining the physiological CO_2_ fixation activity of *Chromatium okenii*, the microorganism responsible for bioconvection. Furthermore, the turbulence generated by bioconvection redistributes sulfides to the upper region of the BL and displaces other microorganisms from their optimal ecological niches.

**Conclusion:**

The findings underscore the influence of bioconvection on the physiology of *C. okenii* and demonstrate its functional role in improving its metabolic advantage over coexisting phototrophic sulfur bacteria. However, additional research is necessary to confirm these results and to unravel the multiscale processes activated by *C. okenii’s* motility mechanisms.

## Introduction

Bioconvection is an organized behavior observed, mainly in laboratory settings, in diverse groups of motile microorganisms, that share a common upward swimming behavior and a density higher than water (Ochiai et al., 2011; Ghorai and Panda, 2013; Abe et al., 2017). The convective motion is triggered when a large number of such microorganisms accumulate in a specific zone of a water body, leading to an increase in local density. This accumulation generates hydrodynamic instabilities with the underlying water, causing the denser, cell-rich layer to sink due to gravity in the form of characteristic ‘plumes’. The bioconvection cycle is sustained by the microorganisms carried away from the sub-surface layer being replaced by others up swimming from below, generating a convective cycle (Hill and Pedley, 2005; Bouffard and Wüest, 2019; Yanaoka and Nishimura, 2022).

Bioconvection can be distinguished from other drivers of water flow, such as wind, tides, and temperature gradients, due to its reliance on motile microorganisms, such as algae or bacteria (Hill and Pedley, 2005; Bees, 2020). It is characterized by the presence of biological concentration gradients within the fluid medium, which are created by the active movement of these microorganisms. Additionally, bioconvection typically occurs on small length and time scales, with a low Reynolds number compared to other forms of fluid flow, involving a dynamic feedback loop where microorganisms respond to the flow and chemical gradients they generate, making it a unique and complex process in aquatic ecosystems (Ochiai et al., 2011; Williams and Bees, 2011; Yanaoka and Nishimura, 2022).

In laboratory, the focus has been on photo- and gyrotaxis (Bees, 2020; Sengupta, 2023), while more recent works have shown that bioconvective movements can increase oxygen and nutrients transfer, providing benefits to microbial communities (Tuval et al., 2005; Tam and Hosoi, 2011; Ardekani et al., 2017). In the natural environment, it has been demonstrated that biotic communities can cause mixing similar to that produced by wind and tides in marine settings (Dewar et al., 2006; Katija and Dabiri, 2009; Lin et al., 2011). Nevertheless, within oceanic environments, the eco-physiological implications stemming from the convective mixing generated by motile microorganisms are yet to be elucidated (Kils, 1993; Kunze et al., 2006). Thus far, the observation of bioconvection in freshwater ecosystems has remained elusive, resulting in a lack of information on the fluid dynamics and energy balance caused by this process.

This research gap persists despite the potential importance of bioconvection for these ecosystems, where its effects may be more conspicuous than in marine environments because of the smaller size of the water bodies.

Meromictic lakes exhibit permanent stratification and vertical gradients of light and redox conditions (Gulati et al., 2017). Their water column harbors several physiological groups of microorganisms distributed along these vertical gradients, serving as distinct ecological niches (Rogozin et al., 2010; Andrei et al., 2015; Gulati et al., 2017; Block et al., 2021).

Lake Cadagno is an example of crenogenic meromixis, a phenomenon occurring when saline springs discharge dense water into a freshwater lake depression, setting up density-stabilizing conditions (Tonolla et al., 2017). As a result, the water column is permanently stratified into an oxic, electrolyte-poor mixolimnion and a salt-rich, sulfidic monimolimnion. Between the two layers, at a depth of about 12.0 m, is an oxidoreductive transition zone, commonly referred to as the chemocline, where the oxygen disappears and sulfide develops (Di Nezio et al., 2021). A characteristic feature of Lake Cadagno is the presence of a community of anoxygenic phototrophic sulfur oxidizing bacteria, which develop due to the euxinic environment and the presence of light (Ravasi et al., 2012; Danza et al., 2018). This community forms a dense bacterial layer (BL; up to 10^7^ cells ml^−1^ in the warm season) and is composed of seven species of purple sulfur bacteria (PSB) and two species of green sulfur bacteria (GSB), which compete for the same resources (light and sulfide). The most representative BL species, accounting for over 80% of the total microbial community (Danza et al., 2018), encompass the large motile PSB *Chromatium okenii* (Luedin et al., 2019a), the small PSB *Thiodictyon syntrophicum* (Peduzzi et al., 2012) and the two GSB species of the genus *Chlorobium*, *C. phaeobacteroides*, and *C. clathratiforme* (Habicht et al., 2011). These three bacterial phenotypes represent different evolutionary strategies, and due to their different sizes, they can be effectively monitored by flow cytometry (Danza et al., 2017). Large-celled *C. okenii* (8.0–10.0 μm) displays active motion through positive phototaxis and negative aerotaxis (Pfennig et al., 1968; Luedin et al., 2019a). Small-celled *T. syntrophicum* (1.4–2.4 μm) has the capacity to create aggregates with microorganisms that produce sulfide, i.e., sulfate-reducing bacteria (SRB) from the genus *Desulfocapsa* (Tonolla et al., 2000). Conversely, the *Chlorobi*, due to their small size (0.6–1.3 μm), have a well-developed pigment apparatus that enables them to harvest lower light intensities (Frigaard and Bryant, 2006; Overmann, 2006). In the course of multiple monitoring efforts carried out on Lake Cadagno, spanning over 30 years of scientific research, the annual population dynamics of the BL have been characterized (Tonolla et al., 2005; Peduzzi et al., 2011; Storelli et al., 2013; Luedin et al., 2019b). During the initial warm season, from June to October, *C. okenii* exhibits dominance, whereas GSB and small-celled PSB *T. syntrophicum* take over the BL community in late August-beginning September (Tonolla et al., 2003; Danza et al., 2018). This population dynamic during the warm season also coincides with the occurrence of bioconvection, observed in Lake Cadagno only between June and late August, based on the physicochemical profiles of the water column, as recently reported in Sommer et al. (2017). Combining field and laboratory studies with numerical modeling, the authors of the study demonstrated that the motile PSB *C. okenii* can initiate bioconvection in the BL of meromictic Lake Cadagno and convectively homogenize a one-meter-scale layer, both in the presence of light and in the dark (Sepúlveda Steiner et al., 2019, 2021). This was the first example of bioconvection witnessed in a natural freshwater environment, so far limited to observations in marine ecosystems or laboratory settings. However, the study by Sommer et al. (2017) focused on the physical demonstration of the phenomenon without exploring its consequences on microenvironmental conditions, microbial community, or biogeochemical implications on the lake ecosystem. Furthermore, the BL community plays a crucial role in the lake’s ecology by participating in central biogeochemical cycles like carbon (Posth et al., 2017), sulfur (Di Nezio et al., 2021) and nitrogen (Philippi et al., 2021).

The objective of this study was to investigate whether the presence of bioconvection in Lake Cadagno during the peak of the warm season (July) significantly impacts the physicochemical profiles of the water column and the eco-physiological characteristics of the anoxygenic phototrophic sulfur bacteria of the BL when compared to the absence of bioconvection (September).

## Methods

### Study site and sampling

Lake Cadagno is located in the Piora Valley (46°33’N, 8°43′E) in the southern Swiss Alps. The sampling was conducted on 16 July and 17 September 2020 using a pump-CTD system (CTD115M, Sea & Sun Technology, Germany), as described in Di Nezio et al. (2021). For the physicochemical characterization of the water column, *in situ* high vertical resolution data (sampling at 16 Hz) on temperature (°C), conductivity (mS cm^−1^), dissolved oxygen (mg l^−1^), light (PPFD) and turbidity (FTU) were obtained during a first continuous downcast of the CTD system from the lake surface down to ~18.0 m depth. During a second downcast, after 30 min and in a different area, discrete water samples were collected from six depths (between 12.0 m and 18.0 m) for bio-chemical analyses and from the top of the BL for incubation experiments (see ‘Radioisotope incubation and uptake analysis’ section). Profiles were recorded at sunrise on 16 July and 17 September 2020 at 05:15 h and 06:30 h, respectively. CTD profiles for determination of light regimes were measured at daytime (17:00 h) on both days.

Atmospheric radiation data at a 10-min resolution were retrieved from a meteorological station (istSOS)^1^ close to the lakeshore during both sampling campaigns. Water samples for microbiological (FISH and flow cytometry) and chemical (S^2−^, SO_4_^2−^ and CaCO_3_) analyses were stored in 50 mL falcon tubes and processed within the following hour, as described in Di Nezio et al. (2021).

### Cell growth

The main characteristics of the phototrophic sulfur bacteria strains used in this study are described in Table 1.

**Table 1:** Main morpho-physiological characteristics of the strains used in this study.

Name	Morpho-physiology	Reference
*Chromatium okenii* strain LaCa	Purple sulfur bacterium; rod-shaped cells, 8.0–10.0 μm in size, Gram-negative; motile through flagella; sulfur assimilated in intracellular sulfur globules; anaerobic or microaerophilic growth; CO_2_ fixation by the Calvin-Benson-Bassham (CBB) cycle	Luedin et al. (2019a)
*Thiodictyon syntrophicum* strain Cad16^T^	Purple sulfur bacterium; round-shaped cells, 1.4–2.4 μm in size, Gram-negative; non-motile, vacuolated; sulfur assimilated in intracellular sulfur globules; anaerobic or microaerophilic growth; CO_2_ fixation by the Calvin-Benson-Bassham (CBB) cycle	Peduzzi et al. (2012)
*Chlorobium phaeobacteroides* strain 1VII D7	Green sulfur bacterium; rod-shaped cells, 0.6–1.3 μm in size, Gram-negative; non-motile; sulfur deposited in extracellular sulfur globules; strictly anaerobic growth; CO_2_ fixation by the reverse tricarboxylic acid (*r*TCA) cycle	Overmann (2006)

PSB and GSB were grown in Pfennig’s medium I (Eichler and Pfennig, 1988) and cultivated in laboratory under a light/dark photoperiod of 16/8 h with a light intensity of 38.9 μmol m^−2^ s^−1^ PPFD (photosynthetic photon flux density) within the photosynthetically active radiation (PAR) range, measured with a portable LI-180 Spectrometer (LI-COR Biosciences, Lincoln, NE), as in Di Nezio et al. (2021).

### Bacterial layer microbial community analysis

To describe the composition of the BL microbial community, cell counting was performed through flow-cytometry (FCM), as described in Danza et al. (2017). Bacterial populations were distinguished by applying gates on cell size (forward scatter, FSC) of 0.6–1.3 μm for GSB, 1.4–4.0 μm for small-celled PSB and 8.0–10.0 μm for *C. okenii* (Supplementary Figure S1). Simultaneously, fluorescent *in situ* hybridization (FISH) analyses were carried out on bacterial layer (BL, zone with a turbidity >10 FTU) water samples, as previously described in Decristophoris et al. (2009) (Supplementary Table S1), spotting 2 μL of fixed sample on gelatin coated slides (0.1% gelatin, 0.01% KCr(SO_4_)_2_) and observing them by epifluorescence microscopy using filter sets F31 (AHF Analysentechnik, Tübingen, Germany; D360/40, 400DCLP, D460/50 for DAPI) and F41 (AHF Analysentechnik, HQ535/50, Q565LP, HQ610/75 for Cy3) at 100X magnification.

### Radioisotope incubation and uptake analysis

To test the photosynthetic efficiency of pure bacterial laboratory cultures and describe their different ecological niches, cells were grown up to concentrations of 10^6^ cells ml^−1^ (mid exponential phase), sealed in 50-cm-long dialysis bags (inflated diameter of 62.8 mm; Karl Roth GmbH Co. KG, Germany), whose membrane allows for diffusive transport of small molecules (< 20 kDa); thus, preventing contamination of the incubated samples from the surrounding environment.

The dialysis bags were acclimatized to the chemocline conditions of Lake Cadagno for a period of 4 weeks (from 15 June to 16 July 2020 and from 21 August to 17 September 2020) before the experiments, at varying depths between 12.20–12.40 m and 13.12–12.94 m in July and September, respectively.

To measure the amount of light reaching the incubation depth, the dialysis bags supporting frame was equipped with HOBO UA-002–64 Pendant passive data loggers (light measurement range: 0 to 320,000 lux; Onset Computer Corporation, MA, USA), one at the top and one at the bottom of the structure, measuring relative light (Lux; 180–1,200 nm) at 60 min intervals during the 4-weeks acclimatization periods, as well as over the course of the 24 h ^14^C incubations (16–17 July and 17–18 September 2020). Average daylight intensity values for June–July and August–September recorded at the top and bottom of the dialysis support frame are shown in Supplementary Figure S2.

The ^14^C-radioisotope uptake experiment was carried out on 16 July and 17 September 2020. The ^14^CO_2_ assimilation of every selected microorganism, was quantified in sealed glass bottles after a day and night incubation, both in July (at 12.28 m and 12.35 m depth) and September (at 12.63 m and 12.83 m depth). The total inorganic carbon assimilation measured on water sampled from the top of the BL served as a positive control of cell activity. The same water was also filtered through 0.22 μm filters to remove any microorganisms and define a zero value to be used as a negative abiotic control.

The dissolved inorganic carbon concentration needed for the calculation (Gächter and Marès, 1979) was determined using the CaCO_3_ Merck Spectroquant kit (No. 1.01758.0001) and the Merck Spectroquant Pharo 100 spectrophotometer (Merck, Schaffhausen, Switzerland). Rates were expressed as attomoles (amol) of ^14^CO_2_ per cell volume per hour. Cell volumes were calculated as described in Tonolla et al. (2003). The total biovolume of the various species in the control sample was computed using the relative abundances quantified by flow cytometry and FISH.

### Light and energy requirements calculations

HOBO light and carbon assimilation data were used to calculate the quantum requirement for the CO_2_ fixation as the ratio between moles of photons absorbed and moles of ^14^CO_2_ fixed by the BL and the dialysis bags pure cultures. Moles were correlated to the surface area (m^2^) of the glass vials used for the ^14^C-incubation to calculate how many moles of photons reached the surface of the vials over the entire light period (Brune, 1989).

### Statistical analysis

Data are shown as mean ± standard deviation. All the measurements were taken from distinct samples. Statistical significance was assessed by two-way ANOVA for parametrical data, as indicated; Šidák test was used as a post-hoc test. For multiple comparisons, multiplicity adjusted *p*-values are indicated in the corresponding figures. Statistical analyses comprising calculation of degrees of freedom were done using GraphPad Prism 9.5.1.

## Results

### Monitoring of the water column

**Figure 1:**
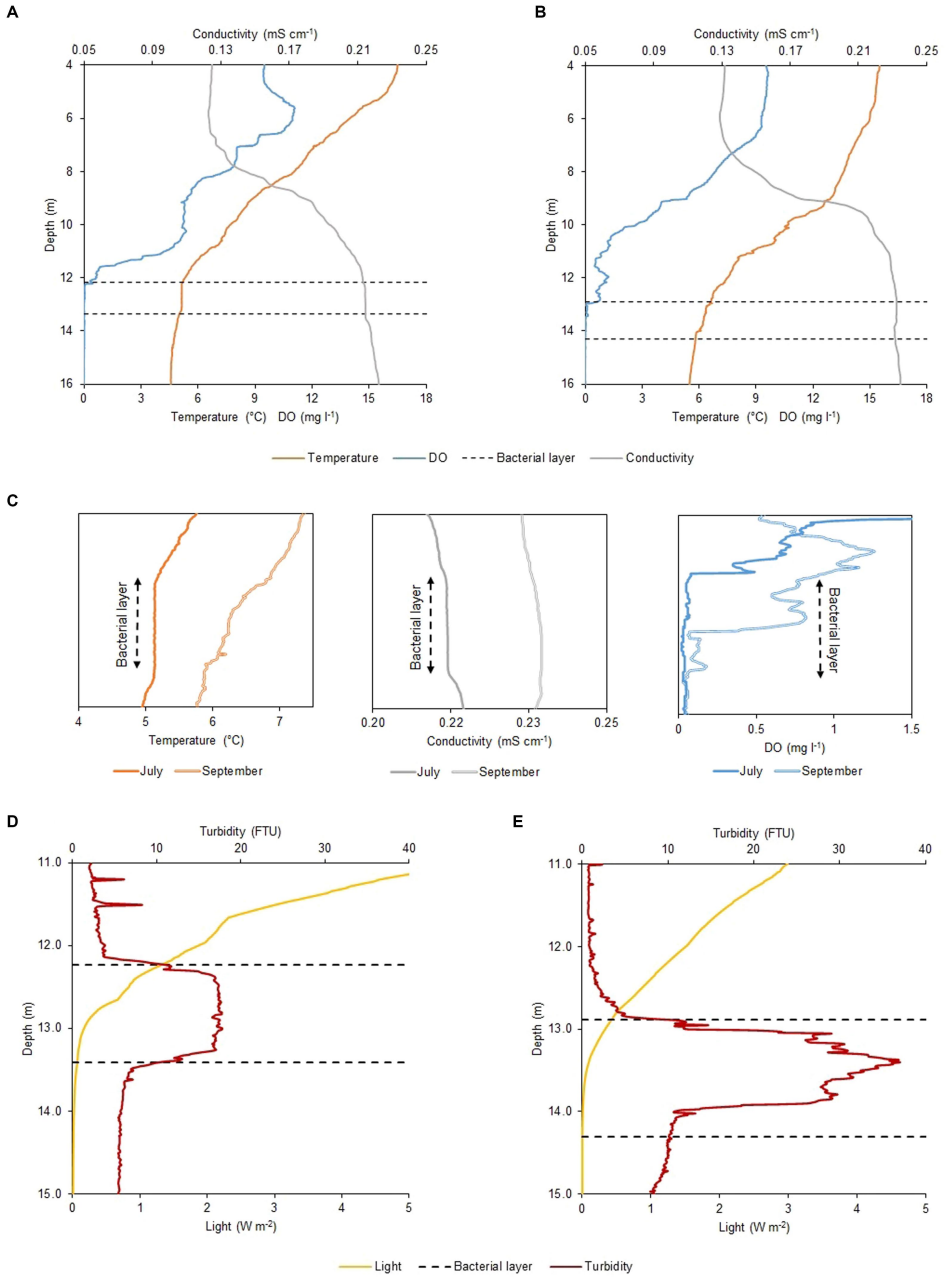
Physicochemical profiles of Lake Cadagno water column. CTD profiles of oxygen (mg l^−1^), temperature (°C), and temperature-corrected (20°C) conductivity (mS cm^−1^) in **(A)** July and **(B)** September. **(C)** Temperature *(left)*, temperature-corrected conductivity *(central)* and DO *(right)* microstructure profiles for the BL and adjacent regions observed on the two different moments of the year 2020. Different light irradiance (W m^−2^) and turbidity (FTU) values within the BL observed between **(D)** July and **(E)** September. Black dashed lines indicate the BL position on both sampling days (16 July and 17 September 2020).

The physicochemical profiles of Lake Cadagno remained largely unchanged between July and September 2020 (Figures 1A,B). Parameters measured with the CTD including dissolved oxygen (DO), temperature, and conductivity showed little difference between the two periods throughout the water column. Nonetheless, upon thorough analysis of the profiles, temperature and conductivity appeared vertically homogenous around 12 m depth in July (as illustrated in Figure 1A), concurrently with the presence of the BL (Figure 1C). Conversely, the profiles observed in September (Figure 1B) did not exhibit such uniformity. The oxygen profile shows little variation between July and September (Figure 1C, right box). In July, oxygen depletes before the top of the BL, whereas in September, it only disappears in the middle of the BL. The dissolved oxygen profile of July exhibits irregularity, notably, a peak in production occurring at 5 m and a minor one at 11 m depth (Figure 1A), indicating the occurrence of oxygenic photosynthesis. The main difference between the July and September physicochemical profiles was observed in the light and turbidity profiles (Figures 1D,E). A turbidity value higher/lower than 10 FTU, is used to define the presence/absence of the BL. On 16 July 2020, the 1.2 m wide BL lied between 12.18 and 13.36 m depth, with a maximum turbidity value of 17.7 FTU at a depth of about 12.70 m (Figure 1D), while on 17 September 2020, it was nearly 20 cm wider (1.4 m) and about 70 cm deeper (12.89–14.30 m depth) with a maximum turbidity peak of 36.9 FTU almost a meter deeper than in July, at 13.40 m (Figure 1E). Such difference in depth resulted in a disparate light profile with an intensity reaching the BL top of 0.44 W m^−2^ in September, twice lower than in July with 0.88 W m^−2^ (Figures 1D,E).

### Differences in light availability

Data collected with the CTD PAR sensor (Figures 1D,E, yellow line) and the pyranometer of the meteorological station (Table 2), revealed a difference in the duration and intensity of light between July and September. The net radiation measurement showed an average photoperiod in July of around 16.0 h (05:15–21:20) while in September it reduced to about 12.5 h (06:40–19:20 h). This resulted in a PAR portion of the average incident radiation below the lake subsurface (1 cm depth) of 100.85 W m^−2^ day^−1^ on 16 July and 43.62 W m^−2^ day^−1^ on 17 September and, consequently, light radiation reaching the top of the BL was higher in July (Table 1). Moreover, in September the larger values of observed turbidity (Figure 1E) determined a greater degree of light attenuation across the BL. Therefore, the shading effect exerted by the turbidity peak ultimately caused no light to reach the lower part of the BL (Table 2).

**Table 2:** Lake Cadagno and BL light regimes (W m^−2^ day^−1^) during the two sampling days.

Date	Light period (h)	Lake subsurface	Top BL	Bottom BL
16.07.20	16.0	100.85	1.44	0.06
17.09.20	12.5	43.62	0.44	0.00

### Sulfide and intracellular complexity at the top of the BL

**Figure 2:**
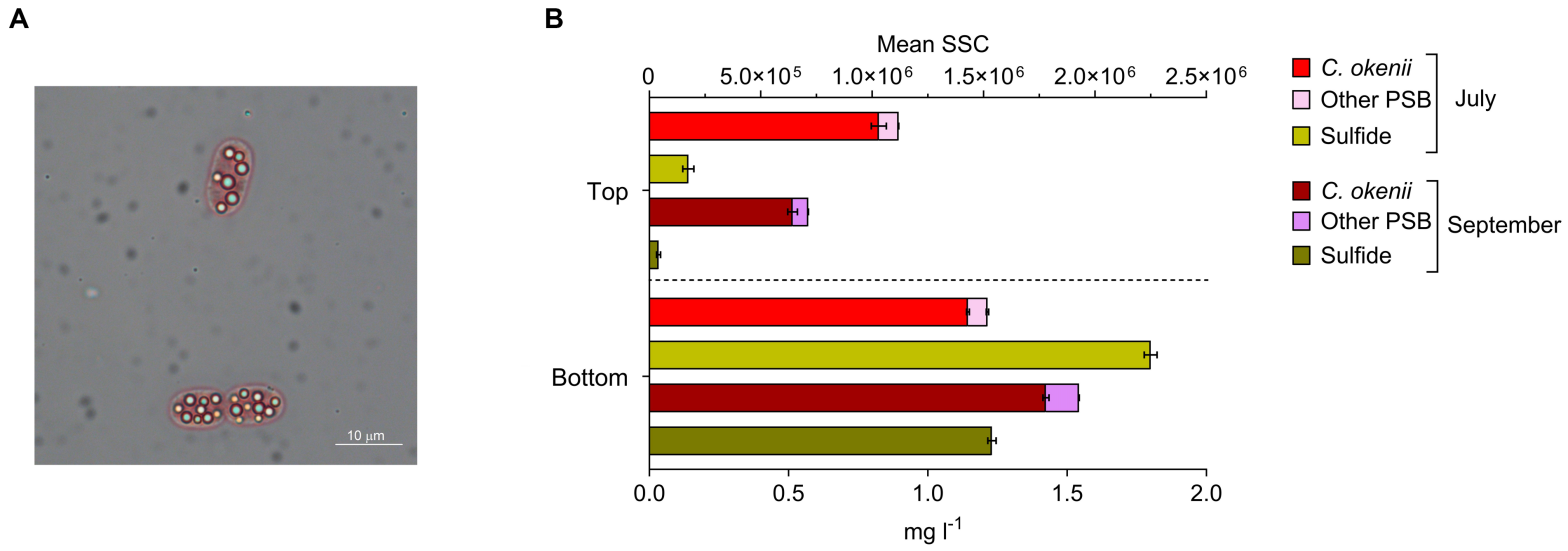
**(A)** Microscopy image of *Chromatium okenii* cells (100X magnification) with intracellular sulfur globules. **(B)** Flow cytometry mean sideward scatter (SSC) of *C. okenii* and small-celled PSB and corresponding sulfide concentrations in the upper and lower part of the bacterial layer on 16 July and 17 September 2020. Error bars represent standard deviation (*n* = 3).

In addition to light, anoxygenic phototrophic sulfur bacteria in the BL require reduced sulfur compounds, such as sulfide (S^2−^) or elemental sulfur (S^0^). These compounds are present in the water after the SRB metabolism, and in the form of characteristic sulfur globules, produced intracellularly by PSB and extracellularly by GSB (Figure 2A). On 16 July, S^2−^ concentration measured at sunrise, before the light reached the BL, was around 0.14 mg l^−1^ at the top of the BL and 1.88 mg l^−1^ at the bottom, while on 17 September, at the same moment of the day, sulfide concentration at the top was zero (below the detection limit of 0.03 mg l^−1^) and 1.23 mg l^−1^ at the bottom (Figure 2B, yellow bars).

The occurrence or lack of sulfide at the top of the BL, where there is still enough light for anoxygenic photosynthesis, shows a positive correlation with cellular complexity (SSC) that is determined by flow cytometry (Danza et al., 2017). In July, when sulfide is present at the top of the BL, we observed a greater intracellular complexity for PSB, with emphasis on *C. okenii* (Figure 2B, red and pink bars). In September, when, despite the lower light, S^2−^ is no longer available, a marked reduction occurred in the SSC value for *C. okenii* (Figure 2B, burgundy and purple bars).

### Differences in the BL community composition

**Figure 3:**
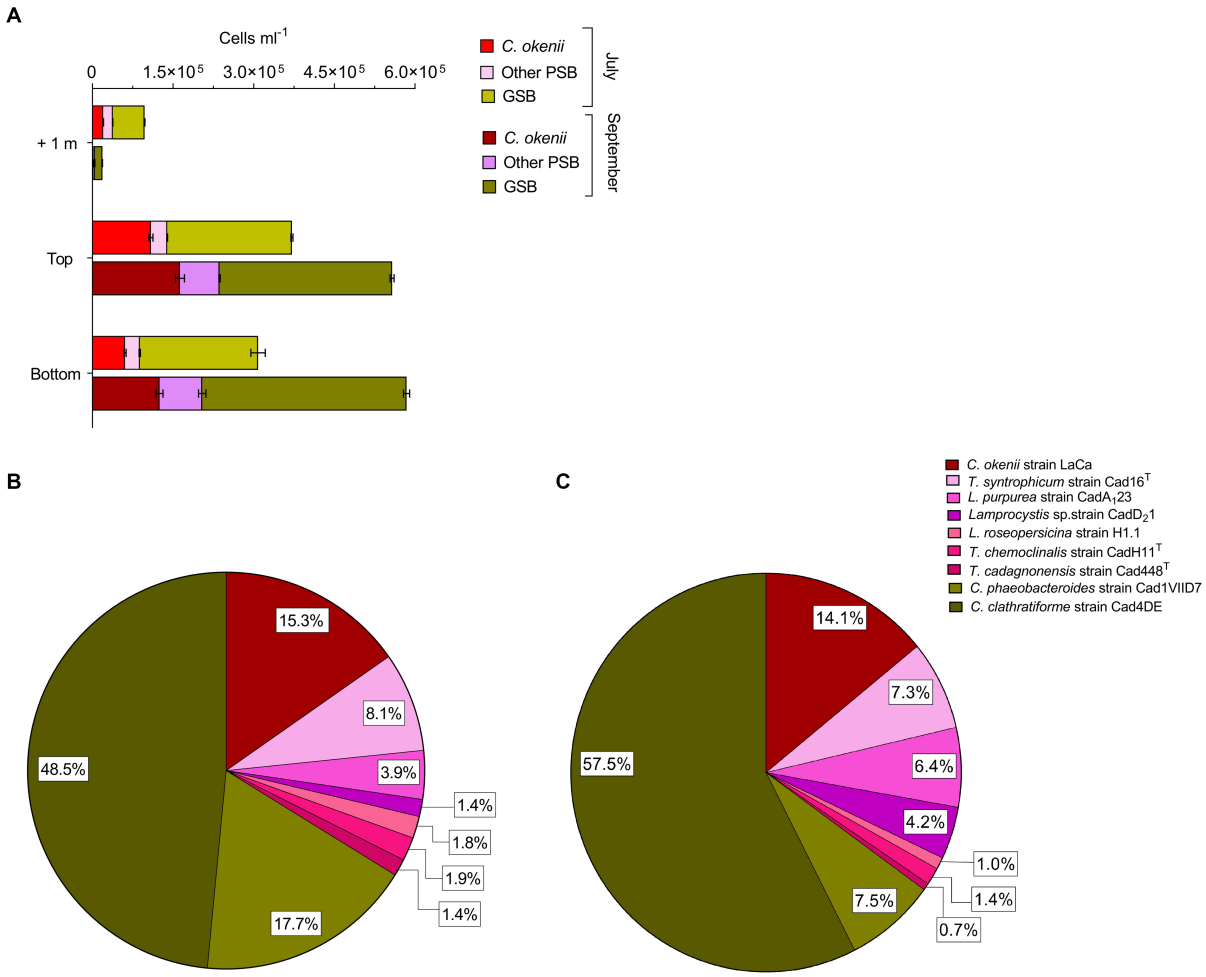
**(A)** Flow cytometry counts revealed different abundances of anoxygenic phototrophic sulfur bacteria 1 m above, at the top and bottom of the BL between 16 July 2020 (light colors) and 17 September 2020 (dark colors). Cell numbers (×10^5^ mL^−1^) of all phototrophic sulfur bacteria in the BL obtained by FISH with probes S453F, S453A, S453E, S453H, S453D, S448, CHLP and CHLC at the top of Lake Cadagno BL on **(B)** 16 July 2020 and **(C)** 17 September 2020. Error bars represent standard deviation (*n* = 3).

The total number of cells of *C. okenii* (Figure 3A, red bars), the other 6 PSB species (Figure 3A, pink bars), and GSB (Figure 3A, green bars) was determined by flow cytometry based on the different cell size (Danza et al., 2017).

During both time periods analyzed, the concentration of large-celled *C. okenii* (approx. Length 8.0–10.0 μm) peaked at the top of the BL, when the turbidity surpassed 10 FTU. FCM red fluorescence signal indicated concentrations of 1.09 ± 0.03 × 10^5^ cells ml^−1^ in July (Figure 3A, red bars ‘Top’) and 1.63 ± 0.08 × 10^5^ cells ml^−1^ in September (Figure 3A, burgundy bars ‘Top’). The relative abundance of *C. okenii* then decreased toward the lower part of the BL, with cell concentrations dropping to 0.61 ± 0.02 × 10^5^ and 1.25 ± 0.06 × 10^5^ cells ml^−1^ in July (Figure 3A, red bars ‘Bottom’) and September (Figure 3A, burgundy bars ‘Bottom’), respectively. In July, no significant differences in the concentration of small-celled PSB and GSB were detected between the top and bottom of the BL (Figure 3A, pink and light green bars). Conversely, during September, more PSB and GSB were detected at the bottom of the BL compared to the top (Figure 3A, purple and dark green bars).

Interestingly, in July during bioconvection, flow cytometry counts revealed high numbers of small-celled PSB and GSB 1 meter above the BL, at a depth well outside their preferred anaerobic zone (Figure 3A, pink and light green bars ‘+1’). Conversely, in September, as bioconvection faded, their numbers above the BL decreased considerably (Figure 3A, purple and dark green bars ‘+1’) even though the number of cells was larger. Flow cytometry is a useful tool for providing an overview of the major populations in the BL, but it lacks the resolution needed to differentiate cells with the same morphology as the 7 small-celled PSB and 2 GSB *Chlorobium* species. In contrast, fluorescent *in situ* hybridization (FISH) allows a precise quantification of the complete spectrum of anoxygenic phototrophic sulfur bacteria species within the BL. This method enabled comparative analysis between samples collected in July (Figure 3B) and September (Figure 3C).

Percentages corresponding to the FISH counts were calculated relative to the total cell population within the BL, allowing for the depiction of variations in species ratios over time. FISH analysis revealed that such increase in small-celled PSB from July to September was mainly due to *Lamprocystis* sp., strains CadA_1_23 and CadD_2_1 which raised from 3.9 to 6.4%, and from 1.4 to 4.2% of the total BL counts, respectively. Among GSB, from July to September, *Chlorobium clathratiforme* presence increased by about 9.0%, with a concomitant drop of *C. phaeobacteroides* of 10.2% (Figures 3B,C, dark and light green). The motile PSB *C. okenii*, showed a slight decrease of 1.2% in September.

### Light-dependent physiological efficiency

The physiological efficiency of the different typology of bacterial strains was assessed by analyzing their primary production over a 24-h period and comparing the results between July and September. We used laboratory-grown pure cultures of the PSB *C. okenii* strain LaCa, the small PSB *T. synthophicum* strain Cad16^T^, and the GSB *C. phaeobacteroides* strain 1VII D7. The cultures were incubated in Lake Cadagno for 4 weeks inside dialysis bags (50 cm length) at different depths corresponding to the top of the BL in July and September (12 m and 13 m, respectively). During the four-week incubation period in the lake, the presence of light was constantly monitored both above and below the dialysis bags (Supplementary Figure S2). At the time of the experiment, the dialysis bag cultures were transferred into clear glass bottles containing radiocarbon 14 and incubated day (~16 h) and night (~8 h) on July 16 and again day (~ 12.5 h) and night (~11.5 h) on September 17, along with a water sample from the top of the BL as a control.

Rates of ^14^CO_2_ assimilation are listed in Table 2. In July, the ^14^CO_2_ assimilated by the control sample after daytime incubation was over four times greater than that observed in September.

Among the individual species incubated in the dialysis bags, the large-celled *C. okenii* exhibited the highest ^14^CO_2_ fixation activity in July, and not in September, when maximum assimilation rate was measured for *T. syntrophicum*. In fact, between July and September, *T. syntrophicum* increased its photosynthetic activity by more than 4 times, while *C. okenii* reduced it by more than 5 times. As observed in a previous study (Di Nezio et al., 2021), the photosynthetic activity of GSB *C. phaeobacteroides* is much lower than that of PSB. Nevertheless, it is noteworthy that its activity experienced a nearly twofold increase in September as compared to July.

In general, the uptake of ^14^CO_2_ at night, in the absence of light, was much lower than the diurnal photoassimilation. Despite a rather pronounced difference in photosynthetic activity, the control showed similar assimilation rates in the absence of light, which were quite high at both time points. Among pure cultures, only *C. okenii* exhibited substantial nocturnal fixation in July, while otherwise, dark assimilation remained consistently low.

## Discussion

To date, it remains uncertain whether bioconvection confers positive eco-physiological advantages upon the responsible organism and/or negative effects on analogous microorganisms within its ecological niche. A handful of laboratory-based experiments using pure cultures have suggested that microbes use bioconvection to their benefit for exploiting nutritional microenvironments and improved nutrient absorption efficiency (Ni et al., 2020; Shoup and Ursell, 2021; Sengupta, 2023). For example, experimental observations and fluid dynamic simulations have shown that formation of bioconvection can facilitate oxygen transport, which may in turn benefit aerobic microbial communities, as observed in suspensions of *Bacillus subtilis* (Tuval et al., 2005).

These experiments underscore the potential advantages bioconvection offers to its producers and, concurrently, to other coexisting microorganisms within the same ecological niche. In the present study, we conducted the first examination of bioconvection in its natural environment, unveiling hitherto unexplored eco-physiological aspects of this phenomenon. This was achieved thanks to the possibility of carrying out field experiments directly on meromictic Lake Cadagno, where we compared two distinct moments of the year, mid-July, when bioconvection is well present in the BL of the lake, and mid-September, when bioconvection is absent (Sommer et al., 2017; Sepúlveda Steiner et al., 2019).

### Chemocline physicochemical parameters

First, we monitored and compared the physicochemical parameters of Lake Cadagno water column on 16 July and 17 September 2020, which were consistent with past measurements of the same periods (Danza et al., 2017; Luedin et al., 2019a; Di Nezio et al., 2021). The weather trend for summer 2020 was within the normal range, with good insolation and little precipitation, with the only exception of a heavy thunderstorm in late August that caused a strong mixing of the mixolimnion, and a consequent increase in light penetration to the BL (Storelli et al., under revision). On 16 July, the water column profile revealed the homogeneous temperature and conductivity signatures within the BL, right below the oxic-anoxic interface of the chemocline (Figure 1C), a proxy of the presence of convective turbulence, which has been shown to be caused by the swimming activity of PSB *C. okenii* (Frigaard and Dahl, 2008; Sommer et al., 2017). Therefore, the lack of a uniform layer in the CTD profile of 17 September confirmed the absence of bioconvection in late Summer, further highlighting its seasonality, as already observed in previous studies on Lake Cadagno (Danza et al., 2017; Barnett et al., 2020).

Data presented by Sommer et al. (2017) showed how, over the course of three summer seasons (August 2014 and 2015, July 2016), bioconvection activity and thickness of the mixed layer in Lake Cadagno positively correlated with *C. okenii* cell concentration. In fact, the authors observed that an increase in the concentration of bioconvecting *C. okenii* cells corresponded with a proportional increase in the thickness of the mixed layer. A higher concentration of *C. okenii* has also been correlated to augmented convective intensity (Sepúlveda Steiner et al., 2021). We refer to the authors’ direct numerical simulations (Figure 2, Sepúlveda Steiner et al., 2021, Supporting Information Text S3 of the paper) for convincing evidence that the homogeneous layer in Lake Cadagno is due to active biogenic mixing. A detailed explanation of the vertical structure of the mixed layer is provided by Sepúlveda Steiner et al. (2019).

Unfortunately, these observations were limited to the time when bioconvection is present. Interestingly, in September, when bioconvection was absent, *C. okenii* cell concentration was higher than in July, when bioconvection was active. This result highlights the complexity of the process driven by the *C. okenii*, seemingly more related to other abiotic or biotic factors investigated in this study, such as light, sulfide or the presence of other phototrophs in the BL, rather than simply to *C. okenii* population size.

### Bioconvection affects sulfide transport across the bacterial layer

The importance of bioconvection in maintaining chemical gradients across the BL, ensuring the constant influx of key elements, has already been proposed in laboratory studies (Bearon and Grünbaum, 2006; Findlay et al., 2017). Interestingly, the higher S^2−^ concentration across the BL observed in July showed that bioconvection transports sulfide from the depths of the lake (Figure 2; Supplementary Figure S3). In addition to carrying more sulfide, an essential requirement for anoxygenic photosynthesis, bioconvection also promotes the removal of oxygen (Figure 1C, right), which is chemically reduced by S^2−^. The presence of oxygen during the photosynthetic activity of anoxygenic phototrophic sulfur bacteria reduces its effectiveness (42). However, previous studies have also shown that, for PSB, the presence of a small amount of oxygen facilitates CO_2_ assimilation in the dark (Berg et al., 2019; Luedin et al., 2019b; Di Nezio et al., 2021).

A distinguishing feature of PSB is the production of intracellular sulfur globules (S^0^) (Frigaard and Dahl, 2008), that contribute to determining cell internal complexity, correlating with the SSC parameter measured with FCM (Danza, 2018). On 16 July at sunrise, when bioconvection was active (Figure 1C), sulfide was detected up to top of the BL, concurrently with a more homogeneous distribution of intracellular complexity between upper and lower BL (Figure 2). Conversely, at sunrise on 17 September, with no mixed layer, no S^2−^ was detected at the top BL and it mostly remained confined to the lower part, in concomitance with a less pronounced PSB cell granularity at the top BL (Figure 2). Given these points, it is evident that bioconvection has the potential to expand the optimal ecological niche by transporting S^2−^ from the bottom, thereby increasing its presence in conjunction with light.

### Light period affects bacterial motility and primary production

The importance of photoperiod in shaping bacterial motility has been investigated in several studies. For example, experiments on the flagellated microalga *Chlamydomonas reinhardtii* (Jin et al., 2020), and other microalgae (Richter et al., 2014), revealed that variations in the light–dark period not only markedly affected cell swimming behavior but also influenced cell orientational and gravitactic transport. Similar trends have been presented by other studies on the influence of photoperiod length on the motility rhythm of swimming microorganisms, with important consequences at the population scale (Fitt and Trench, 1983; Lerch and Cook, 1984; Barnett et al., 2020).

At first the number of cells was identified as a possible factor influencing bioconvection (Sommer et al., 2017). However, our results suggest photoperiod is a key factor for the onset of bioconvection. In fact, under the shorter light period of September (12.5 h) no mixed layer is observed within the BL, despite the number of *C. okenii* cells being higher than in July (Figure 1). Further evidence comes from laboratory experiments, where a reduction in the growth rate of *C. okenii* was observed under a photoperiod of 12/12 h, compared to one of 16/8 h. Interestingly, even after changing light intensity from a chemocline-like value of about 4.0 μmol m^−2^ s^−1^ PPFD to a 10-fold increase (about 40 μmol m^−2^ s^−1^ PPFD), the reduction in growth was maintained under the 12/12 h photoperiod, further emphasizing the importance of light period rather than its intensity (Di Nezio et al., 2023).

Our results indicate that a different length of the light period can significantly impact photosynthesis itself, by determining different light-stimulated rates of inorganic carbon uptake as showed in other photosynthetic microorganisms (Zhou et al., 2015; Johar Gunawan et al., 2018; Xu et al., 2021), and in PSB and GSB in Lake Cadagno as well. It has been observed that CO_2_ fixation in PSB does not occur at a constant rate throughout the day but reaches the highest values in the first hours of light exposure (Storelli et al., 2013), compared with the hours of highest light intensity in the afternoon (Di Nezio et al., 2021). This trend has also been observed in cyanobacteria (Wyman, 1999; Paul et al., 2000). For this reason, adopting whole day and night incubations allowed us to avoid underestimate carbon assimilation activity due to diel cycles.

In this study, the *in situ* daily ^14^C assimilation observed confirmed the strong total inorganic carbon fixation rate in the BL of Lake Cadagno. On 16 July, with 16 h of light, diurnal assimilation was more than three times higher than in September, when values reached only after a daylength of 12.5 h (Table 2). This result is certainly strongly influenced by the physiological activity of *C. okenii*. In fact, their intense fixation activity measured in July was higher than that of *T. syntrophicum* and *C. phaeobacteroides* (Table 3). The situation changed radically in September, when in the absence of bioconvection, *C. okenii* loses dominance over inorganic carbon fixation in favor of the small-celled PSB *T. syntrophicum*.

**Table 3:** July and September ^14^CO_2_ mean assimilation rates (±standard deviation) of the BL microbial community and dialysis bags incubated cultures.

	July		September	
	Day	Night	Day	Night
Bacterial layer	333.1 ± 8.2	66.7 ± 3.3	78.6 ± 2.2	47.6 ± 1.2
*C. okenii*	81.6 ± 7.6	40.5 ± 2.2	15.5 ± 1.0	1.37 ± 0.1
*T. syntrophicum*	62.6 ± 1.0	24.9 ± 2.1	274.3 ± 14.4	17.1 ± 2.0
*C. phaeobacteroides*	10.2 ± 0.8	9.5 ± 1.8	18.2 ± 1.2	3.5 ± 0.7

Values are reported in ^14^CO_2_ amol μm^−3^ h^−1^.

Despite being more abundant (Figure 3), GSB were outcompeted by PSB for the uptake of inorganic carbon. Nonetheless, to sustain their photosynthetic growth, these bacteria can rely on different substrates. In fact, GSB have the capacity, although limited, for assimilating organic carbon compounds, such as acetate or pyruvate (Frigaard et al., 2003; Tang and Blankenship, 2010).

Similarly, the higher dark ^14^C assimilation rates observed in July in the BL and in *C. okenii* pure culture suggests the persistence of bioconvection during nighttime in Lake Cadagno (Sepúlveda Steiner et al., 2021). Several studies have reported that PSB have the ability fix inorganic carbon in the absence of light by aerobic sulfur-dependent chemoautotrophy (Casamayor et al., 2008, 2012; Berg et al., 2019; Luedin et al., 2019b). In September, our results show good night fixation only for the “*ecological control*,” while cultures exhibited comparatively diminished fixation rates. One plausible explanation for this observation could be attributed to the elevated presence of *Lamprocystis* sp. (Figure 3C), which are well-documented for their proficiency in nocturnal assimilation (Musat et al., 2008; Storelli et al., 2013), although this is probably not the only factor. Interestingly, the correlation between the carbon fixation contributions of PSB and GSB and their abundance does not consistently align, as observed in Lake Cadagno (Musat et al., 2008). Recent studies hypothesized that viral infections affecting PSB and GSB, thereby influencing rates of photosynthetic inorganic carbon fixation, may provide an explanation for this observed pattern (Saini et al., 2022; Hesketh-Best et al., 2023).

In addition, quantum requirements of CO_2_ fixation for each of the three species incubated with carbon-14 were calculated as moles of photons required to assimilate a mole of ^14^CO_2_. The value obtained for *C. okenii* in July (10.6) was similar to that reported by Brune (1989), who calculated quantum requirements of 8.5–10.5 quanta per CO_2_ fixed for PSB and 3.3–4.5 for GSB, under optimum laboratory conditions, while *T. syntrophicum* and *C. phaeobacteroides* had much higher requirements (23.4 and 74.9, respectively). Under the September light regime, *C. okenii* performed significantly worse (38.5), while *T. syntrophicum* (13.5) and *C. phaeobacteroides* (39.1) had both lower quantum requirements than in July. Applying what observed in the ^14^C-incubated cultures to the broader environmental context of the lake, our data provide enough evidence to sustain that light regime played a key role, as it provided *C. okenii* cells with the energy required for the onset of bioconvection.

These findings, in combination with the increase in numbers shown by FISH for all species of small-celled PSB (Figure 3B), further indicate that bioconvection could exert a negative influence on other microorganisms competing for the same ecological niche as *C. okenii*.

### Community dynamics in the bacterial layer

The top-bottom small-celled PSB uniform SSC signal in July correlates well with the hypothesis of *C. okenii* dragging along other microorganisms in the BL, namely small-celled PSB and GSB, that are passively moving floating by means of gas vacuoles (Peduzzi et al., 2012; Luedin et al., 2018). Despite the limitations in differentiating among individual species of small-celled PSB, flow cytometry allows to accurately distinguish *C. okenii* from the other PSB due to their different cell size. In fact, using specific gates on FSC as constant signature for identification, SSC values were measured for all events contained in the specific gate of the respective population (Supplementary Figure S1). In fact, small-celled PSB and GSB populations distribution across the BL highlighted some interesting patterns (Figure 3A). Interestingly, while *C. okenii* cells were in general more abundant at the top BL on both times of the season, on 16 July, during active bioconvection, we measured a higher number of small-celled PSB and GSB 1 m above the BL than on 17 September (without bioconvection). This finding underscores a significant fitness advantage for *C. okenii* over other microorganisms competing for resources such as sulfide and light. Indeed, the displacement by bioconvection of many small-celled PSB and GSB from the optimal BL zone in July (+17%), and not in September (Figure 3A), facilitates an expansion of the ecological niche available to *C. okenii*.

The consequences of bioconvection also appear to affect trophic links, such as grazing by zooplankton. A recent study on the predatory activity of the ciliate *Spirostomum teres*, known to feed on PSB (Bolick, 2022), in Lake Cadagno during the years 2020 and 2021 revealed that, in the presence of bioconvection, the amount of *C. okenii* cells ingested by *S. teres* is significantly lower than when bioconvection is absent (Bolick et al., in preparation). All together, these observations suggest the effective ecological advantage that *C. okenii* has in producing bioconvection.

## Conclusion

In this paper, we analyzed biological, chemical, and physical factors to elucidate how bioconvection shape the main eco-physiological traits for the phototrophic sulfur bacteria community inhabiting the BL of meromictic Lake Cadagno. We first report the key role of the photoperiod length by comparing two different period of measurements. Moreover, we showed how the presence of bioconvection contributes to maintaining a sulfide gradient across the BL, thereby promoting oxygen removal. It is also interesting to note that bioconvection should negatively affects the fitness of the other ecological competitors of *C. okenii*, namely small-celled PSB and GSB, present in the BL. Overall, our combined data suggest that *C. okenii* is able to gain a competitive advantage over other non-motile anoxygenic phototrophic sulfur bacteria in the quest for the optimal environmental conditions by producing mixed layers through bioconvection.

Nevertheless, despite this study provides evidence of the eco-physiological effects of bioconvection in an environmental setting, its consequences on the microenvironmental conditions and the other (micro)organisms involved need to be substantiated with further studies. In particular, the role of bioconvection on the transport of sulfide across the BL, and further insights on its production by sulfate-reducing bacteria in the monimolimnion, require a more detailed investigation. Impacts of bioconvection might also extend outside the mixed layer, influencing the interaction between phototrophic sulfur bacteria and the zooplankton living just above the bacterial layer, e.g., in terms of predation and/or distribution patterns. Lastly, a deeper understanding of the motility mechanisms of *C. okenii* triggering bioconvection at the single-cell level will help unraveling the nature of this multi-scale process.

## Data availability statement

The raw data supporting the conclusions of this article will be made available by the authors, without undue reservation.

## Author contributions

FN, SR, AB-D, and NS designed research and collected samples. FN and SR performed laboratory work. FN, SR, OS, and AB-D analyzed data. FN and NS wrote the paper. All authors developed the concepts and hypotheses covered in this work, and have contributed to the revisions of the manuscript.

## Footnotes

^1^ https://hydromet.supsi.ch/
