# Supplementary material for: Motile bacteria leverage bioconvection for eco-physiological benefits in a natural aquatic environment
Source: Front Microbiol. 2023 Dec 13;14:1253009. doi: 10.3389/fmicb.2023.1253009 (PMC10756677; doi:10.3389/fmicb.2023.1253009)
Supplement: Supplementary file 1 [file Data_Sheet_1.docx]

Supplementary Material


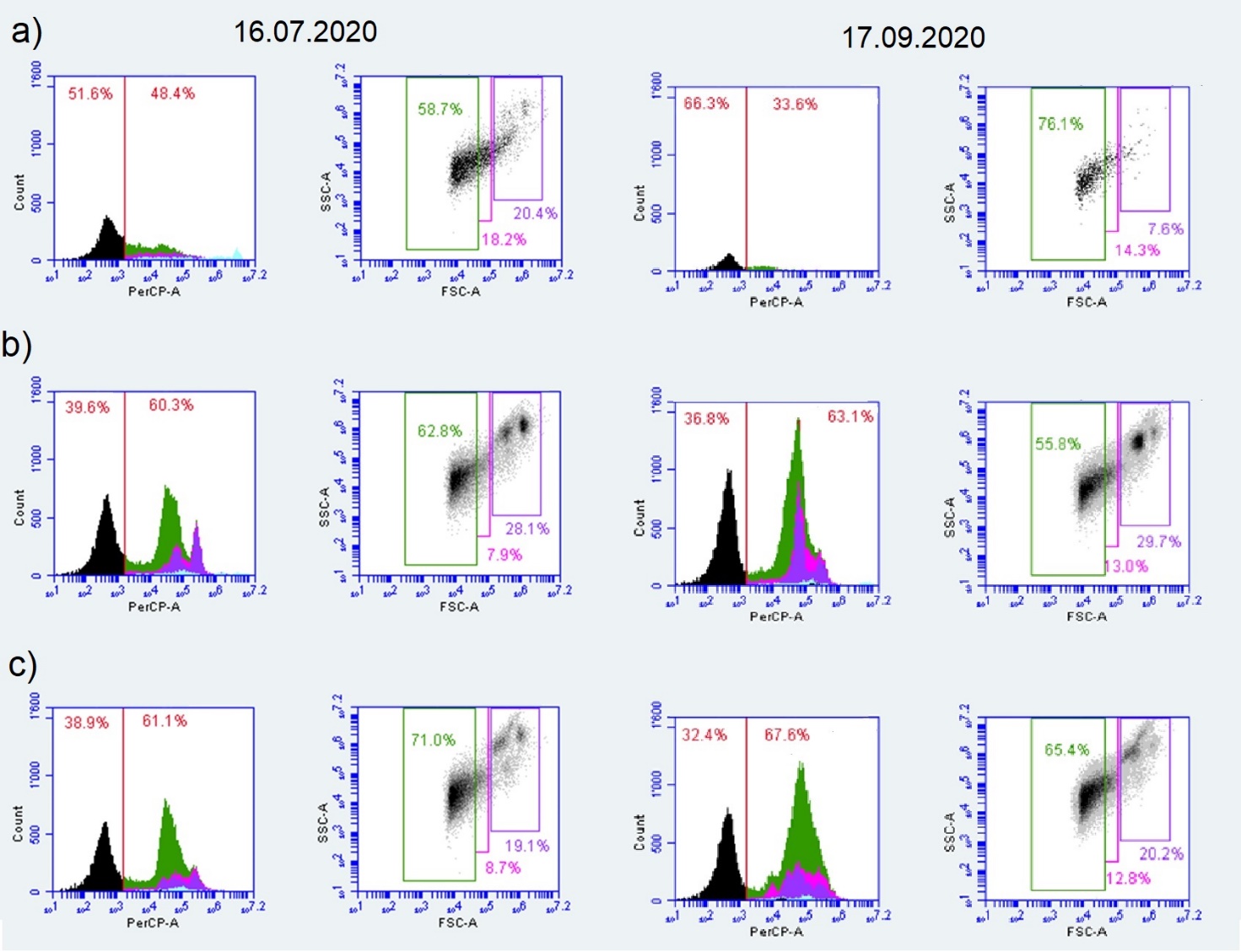


Supplementary figure 1 Flow-cytometry identification and relative abundances of phototrophic populations at a) 1 m above, b) top and c) bottom bacterial layer on 16 July and 17 September 2020. For each date: (left) histograms count vs red fluorescence (logarithmic), red line (PerCP-A > 1 100) separates chlorophyll-pigmented cells from non-autofluorescent cells with relative percentages of the total counts; (right) scatter plots SSC vs FSC with relative percentages of the total chlorophyll-pigmented cells. Purple color identifies PSB *C. okenii* population (8.0 – 10.0 μm), pink defines small-celled PSB (1.4 – 4.0 μm) and green refers to GSB cells (0.6 – 1.3 μm).


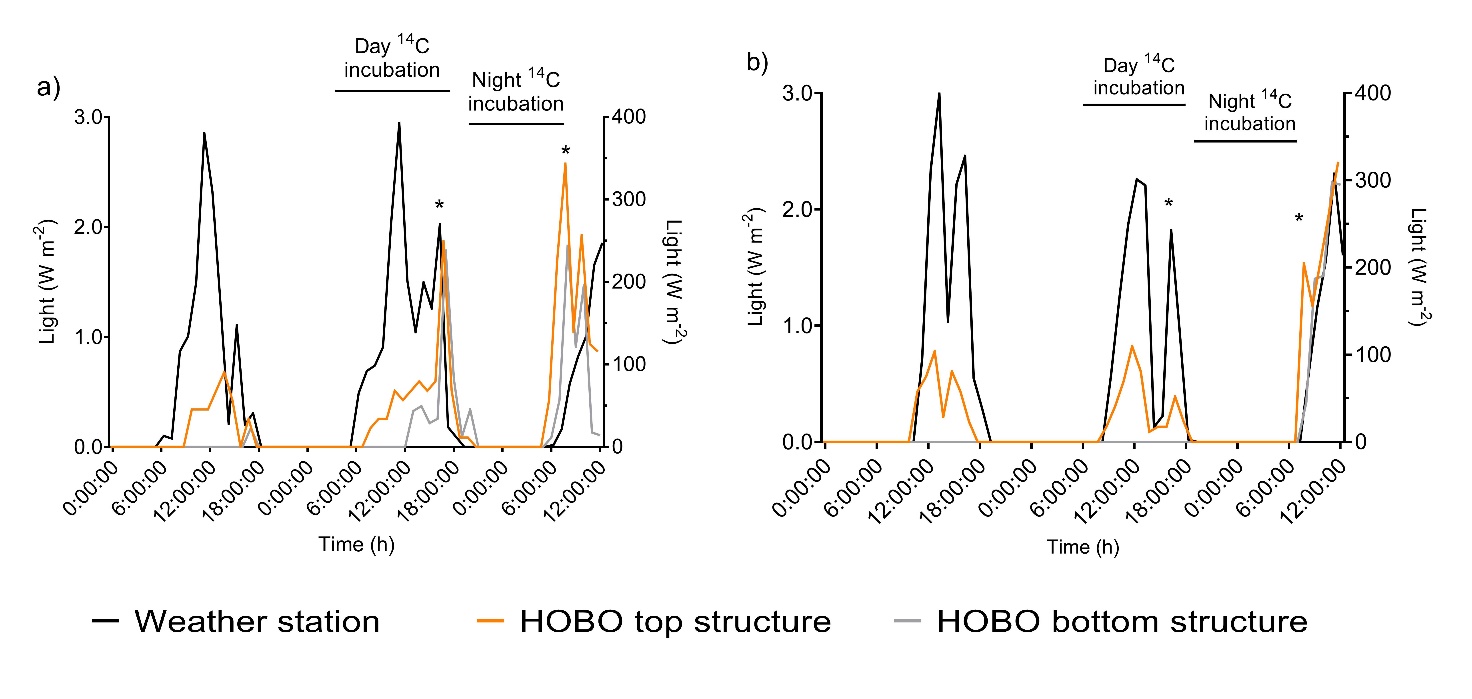


Supplementary figure 2 Net radiation intensity recorded by the weather station and HOBO loggers attached at the top and bottom of the dialysis bags structure during a) 15-17 July 2020 and b) 16-18 September 2020. Asterisks indicate the moment the structure was retrieved at the end of the ^14^C incubation periods (corresponding HOBO light values divided by 100).


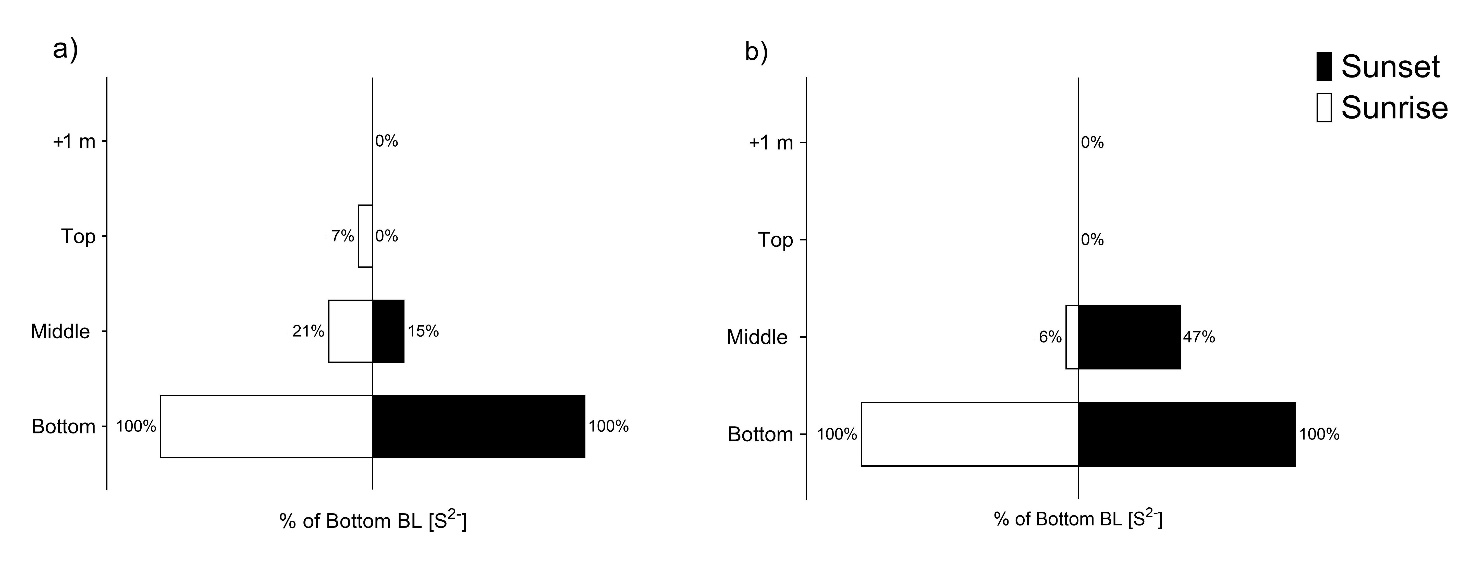


Supplementary figure 3 Sulfide concentration across the BL expressed as percentage of the concentration at the bottom, at sunrise and sunset on a) 16 July 2020 and b) 17 September 2020.

Supplementary table 1 Oligonucleotide probes for the *in-situ* hybridization assay (FISH).

| **Probe** | **Target** | **Sequence** | **Formamide %** |
| --- | --- | --- | --- |
| Cmok453^40^ | *Chromatium okenii* (DSM 169) 16S rRNA, pos.453-479 | AGCCGATGGGTATTAACCACCAGGTT | 30 |
| S453F^40^ | *Thiodyction synthrophicum* 16S rRNA, pos. 453-479 | CGGCGTCGCTGCGTCAGG | 40 |
| S453A^40^ | *Lamprocystis purpurea* (DSM 4197) 16S rRNA, pos. 453-479 | TCGCCCAGGGTATTATCCCAAACGAC | 40 |
| S453E^40^ | *Lamprocystis roseopersicina* (DSM 229) 16S rRNA, pos. 453-479 | CATTCCAGGGTATTAACCCAAAATGC | 30 |
| S453H^19^ | *Thiocystis chemoclinalis* 16S rRNA, pos. 453-478 | GACGGAACGGTATTAACGCCCCGCTT | 10 |
| S448^19^ | *Thiocystis cadagnonensis* 16S rRNA, pos. 448-468 | CGGCGTCGCTGCGTCAGG | 25 |
| S453D^40^ | Clone 261 from Lake Cadagno 16S rRNA, pos. 453-479 | CAGCCCAGGGTATTAACCCAAGCCGC | 40 |
| CHLP^41^ | *Chlorobium phaeobacteroides* (DSM 266), 16S rRNA, pos. 441-464 | AAATCGGGATATTCTTCCTCCAC | 20 |
| CHLC^19^ | *Chlorobium clathratiforme* (DSM 5477), 16S rRNA, pos. 190-211 | GGCAGAACAACCATGCGATTGT | 20 |
| DSC441 + DSC213^42^ | *Desulfocapsa thiozymogenes*   213-230 441-459 | ATTACACTTCTTCCCATCC (DSC441)  CCTCCCTGTACGATAGCT (DSC213) |  |
